# Electrochemical Fabrication of Nanoparticles and Single‐Atom Catalysts via Cathodic Corrosion

**DOI:** 10.1002/chem.202500036

**Published:** 2025-03-17

**Authors:** Mohamed M. Elnagar, Ludwig A. Kibler, Timo Jacob

**Affiliations:** ^1^ Institute of Electrochemistry Ulm University 89069 Ulm Germany

**Keywords:** Electrochemical fabrication, alternating current, square wave potential, nanoparticles, single-atom catalysts

## Abstract

While cathodic corrosion may appear as an undesired degradation process at electrode surfaces, it has become a powerful electrochemical method for fabricating nanoparticles and single‐atom catalysts. In contrast to traditional wet chemical synthesis, cathodic corrosion affords rapid, straightforward, capping‐agent‐free production of nanoparticles, enabling fine control over size, shape, and elemental composition. This mini‐review summarizes recent advances in cathodic corrosion‐based synthesis, emphasizing its unique capabilities for producing metallic, alloyed, and oxide nanoparticles, as well as single‐atom catalysts. It explores the effects of varying parameters such as electrode material, electrolyte composition, voltage waveform, and frequency on the characteristics of the generated particles. Furthermore, it highlights the enhanced electrocatalytic or photoelectrocatalytic performance of the nanoparticles produced via cathodic corrosion.

## Graphical abstract

While cathodic corrosion can be detrimental to electrode surfaces, it has emerged as a powerful electrochemical method for fabricating nanoparticles and single‐atom catalysts from bulk wires. This mini‐review highlights recent advancements in cathodic corrosion‐based synthesis, focusing on its unique capabilities and the superior (photo)electrocatalytic performance of the resulting nanomaterials.

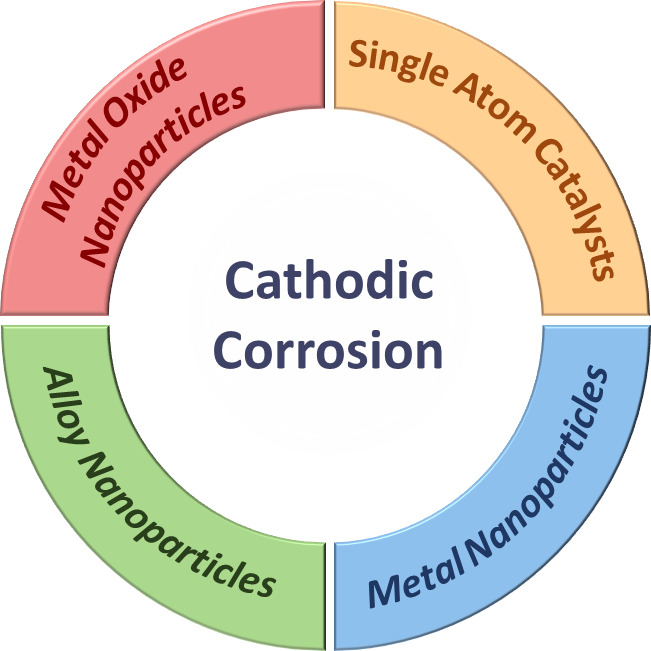

## Introduction

Nanotechnology continues to revolutionize various technological fields such as (electro)catalysis, energy conversion and storage, electronics, (quantum) optics, environmental remediation, and sensors, driven by the unique properties of metal nanoparticles (NPs) and nanostructured materials.[^1^, ^2^, ^3^, ^4^, ^5^, ^6^, ^7^, ^8^, ^9^, ^10^, ^11^] The properties of metal NPs are significantly influenced by their physicochemical features, including size, shape, and composition.[^12^, ^13^, ^14^, ^15^] For bimetallic or multicomponent NPs, these properties are further affected by the elemental distribution within a particle and at its surface, as seen in alloys and core–shell nanoparticles.[^16^, ^17^] Tailoring these parameters allows for the systematic tuning of properties to meet the specific demands of various applications.

The synthesis of metal NPs is generally categorized as “dry” or “wet” methods, with wet chemical approaches providing greater control over particle size and shape.[^18^, ^19^, ^20^] Conventional wet chemical methods typically involve reducing a metal salt precursor in the presence of capping and stabilizing agents, a process that enables the production of metal NPs with controlled shape, size, and composition.[^21^, ^22^] Despite their versatility, these methods often face several limitations such as complex and multistep processes that can hamper reproducibility. Additionally, because the synthesis is often conducted in dilute solutions, they produce low‐concentration NP suspensions, posing challenges for up‐scaling. The need for purification to remove excess surfactants and unreacted precursors adds further complexity.[^19^, ^20^, ^21^]

On that front, electrochemical methods – particularly **cathodic corrosion**, a recent advancement in nanoparticle synthesis – provide promising alternatives to address these challenges. Cathodic corrosion is considered a top‐down strategy that can effectively produce milligrams of nanoparticles within minutes or hours. This process utilizes a simple voltage source and does not require capping agents that might affect catalytic performance.[^23^, ^24^, ^25^, ^26^, ^27^, ^28^]

The phenomenon of cathodic corrosion was discovered more than a century ago when Fritz Haber observed clouds dispersing from negatively polarized metals.[^29^, ^30^] Haber attributed these clouds to the formation and succeeding destruction of alloys between the polarized metal and alkali metal cations. Afterwards, cathodic corrosion was shortly reconsidered in the mid‐1900s.[^31^, ^32^] Nevertheless, it remained mostly an empirical observation during the 20^th^ century and only received growing attention in recent years.[^23^, ^25^, ^33^, ^34^, ^35^, ^36^, ^37^, ^38^, ^39^, ^40^, ^41^, ^42^, ^43^] Cathodic corrosion is an ambiguous electrochemical etching process that induces notable alterations in the surface structure of metal electrodes when exposed to sufficiently negative potentials, particularly in the presence of non‐reducible cations such as alkali metal cations. This phenomenon typically occurs at potentials below the onset potential of the hydrogen evolution reaction.[^23^, ^34^, ^35^, ^36^, ^42^, ^44^] As a consequence of cathodic corrosion, the surface of the cathodically corroded wires is marked by structural transformations and distinct etching features including the formation of etching pits and/or nanoparticles. Interestingly, the combination of cathodic and anodic corrosion has proven to be an effective technique for producing a variety of nanoparticles, including metallic, alloyed, and metal oxide nanoparticles, as well as single‐atom catalysts.[^24^, ^27^, ^28^, ^45^, ^46^, ^47^] Currently, there is a notable scarcity of reviews focusing on cathodic corrosion for nanoparticle production, reinforcing the need to address this gap.

In this mini‐review, we explore recent advancements in cathodic corrosion, highlighting how this method contributes to the efficient fabrication of functional metallic, alloyed, and metal oxide nanoparticles, as well as single‐atom catalysts. We will also discuss the electrocatalytic activity of these nanoparticles and single‐atom catalysts. By examining these developments, we aim to underscore the transformative potential of electrochemical synthesis in advancing nanotechnology for a wide range of scientific and industrial applications.

### Proposed Mechanism Of Cathodic Corrosion

Cathodic corrosion occurs when a metal wire is immersed in an electrolyte containing non‐reducible cations and polarized in the hydrogen evolution regime, *i. e*. at negative electrode potentials, leading to a high coverage of adsorbed hydrogen. In our previous study, we highlighted the crucial role of water and the hydrogen evolution reaction in enabling cathodic corrosion.^[34]^ Additionally, specific adsorption of cations at high coverage is expected at very low potentials.[^36^, ^48^]

During cathodic polarization, short‐lived anionic metal species form and are stabilized by electrolyte cations. These species migrate away from the electrode and encounter water molecules, which are reduced to molecular hydrogen (H₂), while the anionic metal species oxidizes back to its neutral metallic state. The resulting metal atoms can either redeposit onto the electrode or nucleate in solution, leading to nanoparticle formation.[^23^, ^27^, ^28^] Applying alternating currents (AC) enhances cathodic corrosion by introducing periodic anodic corrosion steps, which facilitate nanoparticle detachment from the electrode.[^43^, ^49^]

### Parameters Influencing Cathodic Corrosion

Cathodic corrosion is a simple yet effective electrochemical method for breaking down bulk metals or alloys into highly dispersed nanoparticles or even single atoms. This process involves applying strong cathodic currents to electrochemically etch the working electrode in a conductive electrolyte. A wide range of experimental parameters influence the shape, size, and composition of the produced NPs by cathodic corrosion such as the electrode material, the nature and concentration of cations, alternating current voltages, waveform, frequency, and direct current offset.[^24^, ^28^, ^43^, ^49^] The following section outlines these key factors and provides insights into their interdependent effects on nanoparticle formation:

## 1 Electrode Material

The composition and structure of the electrode play a key role in nanoparticle formation. Pure metals such as Pt, Au, Ag, and Cu exhibit distinct corrosion behaviors compared to alloys and metal oxides. Studies have shown that:

- Pure metals typically form monodisperse nanoparticles, while alloys may experience compositional segregation due to differences in the corrosion rates of individual elements and local surface segregation effects. Despite this, alloys generally retain their bulk composition in the nanoparticles fabricated by this method.[^26^, ^27^, ^28^]
- Electrode pre‐treatment (*e. g*., hydrogen embrittlement in Pd electrodes) can enhance corrosion efficiency and yield more uniform nanoparticles.^[50]^

## 2 Electrolyte Composition

Alkali metal comprised electrolytes (*A*OH, where *A*=Li, Na, K, or Cs) are crucial to trigger cathodic corrosion, but NPs formation also occurred in Ca(NO_3_)_2_, Na_2_SO_4_, CaCl_2_, and NH_4_^+^ cations containing electrolytes.[^27^, ^51^] Notably, cathodic corrosion of metals occurs in highly concentrated alkali metal electrolytes. The high near‐surface concentration of cations is considered essential for cathodic corrosion, as it provides the necessary countercharges to achieve highly negative potentials.^[36]^ Furthermore, higher concentrations of alkali hydroxides promote more aggressive etching and faster nanoparticle production. While cathodic corrosion is often performed without surfactants, the use of surfactants or stabilizers can influence nanoparticle dispersity and shape.^[27]^

## 3 Voltage Waveform and Frequency

The applied potential waveform significantly affects nanoparticle generation efficiency and final properties, with the rate of nanoparticle dispersion strongly influenced by potential perturbations:[^24^, ^28^, ^43^, ^49^]

- Direct current (DC) corrosion tends to produce larger particles due to continuous growth before detachment.
- Alternating current (AC) and square wave potentials (SWP) enhance nanoparticle dispersion, as the positive half‐cycle of the AC voltage weakens the attachment of nanoparticles to the electrode, allowing them to escape into the bulk solution.
- Frequency: Higher frequencies (*e. g*., 1000 Hz) promote finer particle dispersions by limiting the time available for cluster coalescence).[^45^, ^52^]

Given the interplay between these factors, optimization strategies involve:

- Systematic variation of electrolyte composition and voltage waveform to achieve target nanoparticle properties.
- Combining electrode pre‐treatment (*e. g*., annealing or hydrogen charging) with controlled electrochemical parameters.
- Exploring new electrolyte additives that stabilize metal clusters for improved size control.

In the following sections, we demonstrate various electrochemical systems that use cathodic corrosion to fabricate metallic, alloyed, and metal oxide nanoparticles. Furthermore, we discuss examples where cathodic corrosion has been employed to produce highly dispersed single‐atom catalysts. By presenting a variety of electrochemical systems and the experimental parameters optimized for each, insights are provided into the factors influencing nanoparticle characteristics such as size, morphology, dispersity, and composition.

## Synthesis of Metallic and Alloyed Nanoparticles

**Figure 1:**
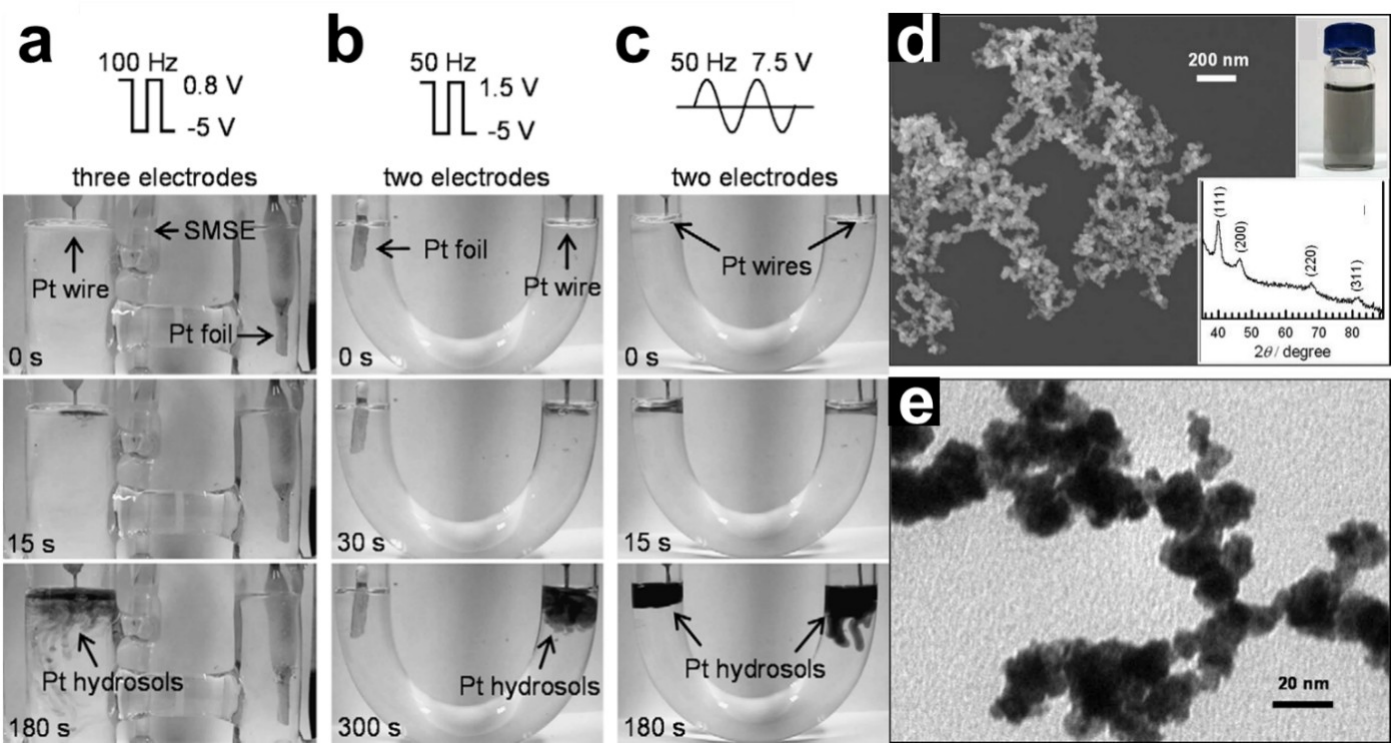
(a–c) Photographic sequence for the production of Pt hydrosols by breaking down a Pt wire in 4 M NaOH through three different experimental procedures. (c) SEM and (d) TEM images for the Pt NPs prepared in 2 M NaOH by SWP (−5 to 0.8 V, 100 Hz). The insets (d) show the optical image of the newly synthesized Pt hydrosols and the XRD patterns of Pt NPs. Adapted with permission from ref.^[43]^ Copyright (2008) Elsevier.

Earlier studies on cathodic corrosion have demonstrated the effectiveness of cathodic corrosion in producing suspended Pt hydrosols by dispersing a bulk Pt wire in a NaOH solution under potential perturbations (either square wave potential (SWP) or alternating voltage (AV)).^[43]^ Figures 1a–c illustrate the time‐dependent formation of Pt hydrosols in 4 M NaOH solution using three different experimental setups. In the first configuration, a three‐electrode system applies an SWP ranging from 0.8 to −5 V at 100 Hz to a Pt wire. Within 15 seconds, a thin grey layer of Pt hydrosols forms around the wire, gradually becoming denser as the dispersion process progresses (Figure 1a). The second setup utilizes a simpler two‐electrode cell, operating under an SWP of 1.5 to −5 V at 50 Hz (Figure 1b). In this configuration, one of the electrodes is a large Pt foil, which experiences a much lower current density and, thus, is less polarized by the perturbations of SWP. The third configuration employs paired electrolysis with an AV of 7.5 V at 50 Hz, applied to two Pt wires in a U‐shaped tube powered by an AV transformer. This setup simplifies the process, generating Pt hydrosols rapidly and efficiently, as both wires simultaneously contribute to nanoparticle dispersion (Figure 1c). Collectively, these configurations demonstrate that the rate of Pt dispersion depends strongly on the potential perturbations. Figures 1d and e show the scanning electron microscopy (SEM) and transmission electron microscopy (TEM) images of Pt nanoparticles synthesized in 2 M NaOH with a square wave potential (0.8 to −5 V at 100 Hz), respectively. The SEM image reveals that the Pt nanoparticles are about 10 nm in size and appear to be aggregates of even smaller particles, as observed in the TEM image. Additionally, the X‐ray diffraction (XRD) pattern confirms the crystalline structure of Pt, with peaks corresponding to characteristic planes in the Pt lattice (inset of Figure 1d). These Pt nanoparticles demonstrated enhanced current density (three times higher) for ethanol oxidation in 1 M NaOH compared to a smooth Pt disk, indicating significantly enhanced electrocatalytic activity. The authors proposed a mechanism for Pt nanoparticle formation under potential perturbation conditions, whereby repeated anodic and cathodic cycles lead to nanoparticle dispersion. In this mechanism, anodic polarization oxidizes surface Pt atoms, forming oxide layers (either monolayer or multilayer). When the electrode is then subjected to highly cathodic polarization, these oxide layers are reduced back to metallic Pt, a process accompanied by substantial hydrogen evolution. The rapid and repeated redox cycling disrupts the freshly reduced Pt atoms, causing them to aggregate into clusters that are then detached from the electrode surface by the force of hydrogen gas evolution, dispersing as nanoparticles into the surrounding solution.

Cathodic corrosion has been also applied for the production of a wide variety of metallic nanoparticles, including Pt, Au, Cu, Ag, Ni, and Rh.^[24]^ Furthermore, the synthesis conditions were fine‐tuned using AC or DC voltages. In the AC setup, a voltage of several volts peak‐to‐peak was applied to the metal electrode, with shorter decomposition times observed at increasingly cathodic potentials. When the AC voltage was offset to include cathodic polarization throughout the cycle, nanoparticle formation continued consistently, with rapid decomposition into a suspension within minutes. The produced Pt NPs showed superior electrocatalytic activity towards carbon monoxide and methanol oxidation reactions compared to commercial platinum catalysts. The enhanced electrocatalytic activity was attributed to the clean surface and high density of low‐coordinated sites produced by cathodic corrosion. In this study, an alternative mechanism was proposed to explain metal nanoparticle formation under high cathodic potentials. According to this mechanism, the process begins with the formation of a water‐free, high‐pH layer at the interface between the metal electrode and solution. Within this alkaline environment, the metal is reduced to anionic species, which are stabilized by the presence of cations. When these anionic metal species come into contact with free water molecules or oxidative agents, they are rapidly reoxidized to their metallic state. The newly formed metal atoms then aggregate to generate nanoparticles. This mechanism highlights the importance of the formation of anionic species and the presence of cations for triggering cathodic corrosion, and thus nanoparticle formation.

A follow‐up study showed the synthesis of size‐controlled platinum nanoparticles through cathodic corrosion in 5 M NaOH or KOH electrolytes using an AC voltage of −10 to 10 V peak amplitude, provided by a non‐inductive power amplifier.^[49]^ By varying the current density during synthesis, the size of produced Pt nanoparticles could be controlled with particle sizes ranging from 6.5 to 12.5 nm. An almost linear relation between current density and particle size was observed, allowing for precise control over nanoparticle dimensions, which is crucial for catalytic applications. The study of electrocatalytic CO oxidation, a highly structure‐sensitive surface reaction, revealed that smaller Pt nanoparticles tend to be poly‐oriented, while larger nanoparticles exhibit CO oxidation activity characteristic of extended Pt(100) terraces.^[53]^ Additionally, comparing synthesis in NaOH and KOH solutions points to possible strategies for further tuning particle size and surface structure, underscoring the adaptability of cathodic corrosion for producing electrocatalysts with tailored properties.

**Figure 2:**
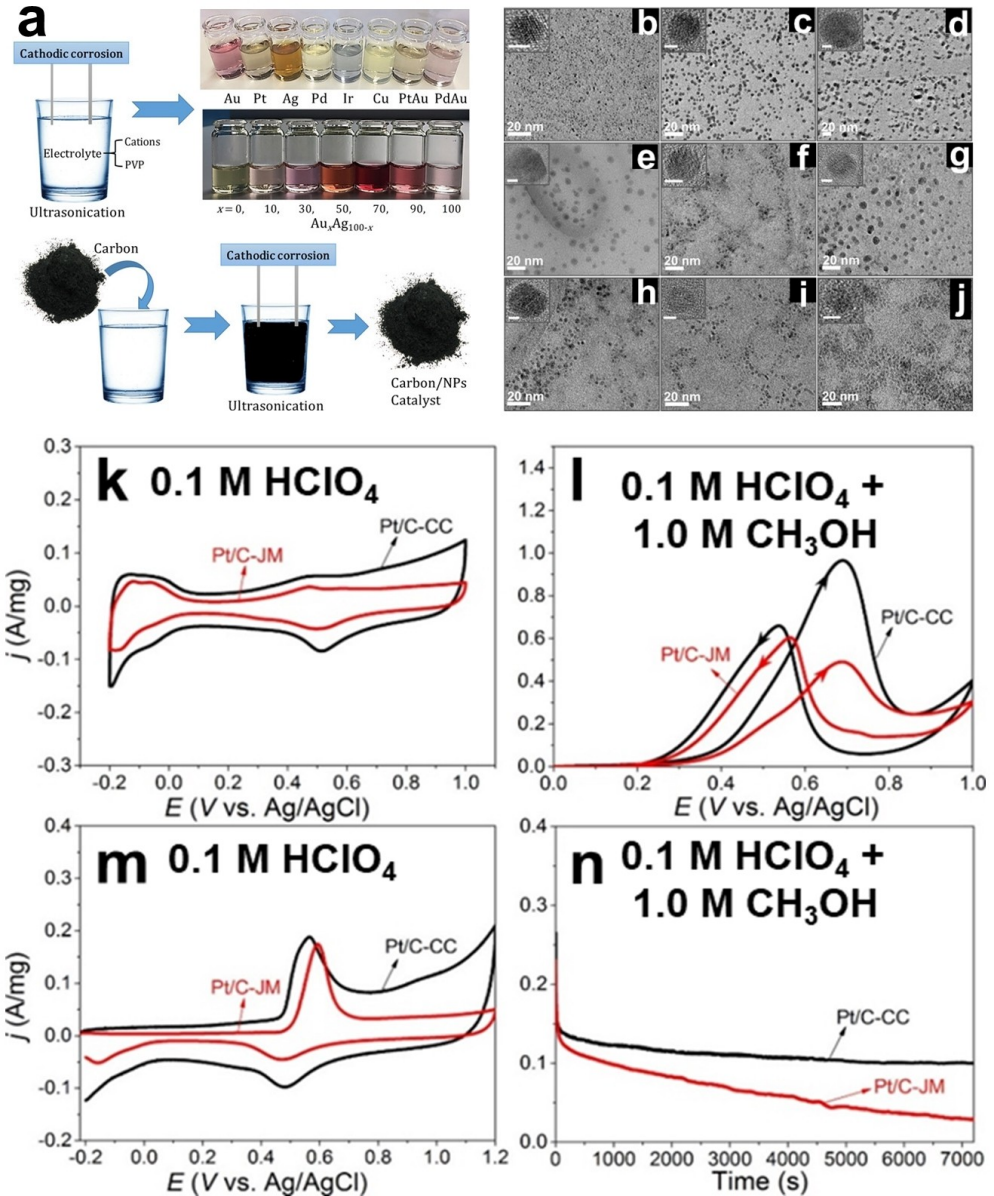
(a) Schematic illustration of the cathodic corrosion setup and the produced colloidal metal nanoparticles. TEM/HRTEM images of (b) Pt NPs synthesized in 0.1 M NaOH, (c) Au NPs obtained in 1 M Ca(NO_3_)_2_, designated as(Au−I), (d) Au NPs prepared in 0.3 M Na_2_SO_4,_ referred as (Au‐II), (e) Ag NPs produced in 0.1 M NaOH, (f) Pd NPs synthesized in 0.3 M CaCl_2_, (g) Rh NPs produced in 0.3 M CaCl_2_, (h) Ir NPs achieved in 0.3 M CaCl_2_, (i) Cu NPs produced in 0.3 M CaCl_2_, and (j) Ni NPs formed in 0.3 M Na_2_SO_4_. All of the bulk wires were treated at ±10 V with a square waveform at a frequency of 100 Hz and were submerged in different electrolyte solutions with 5 wt % PVP. All of the insets in the upper left corner have a scale bar of 2 nm, whereas all of the main Figures have a scale bar of 20 nm. Cyclic voltammograms of Pt/C‐cathodic corrosion and commercial Pt/C electrocatalysts in (k) 0.1 M HClO_4_ at a scan rate of 50 mV s^−1^ and (l) in 0.1 M HClO_4_ + 1.0 M CH_3_OH at a scan rate of 20 mV s^−1^. (m) CO stripping voltammograms of Pt/C‐cathodic corrosion and commercial Pt/C in 0.1 M HClO_4_ at a scan rate of 50 mV s^−1^. (n) Chronoamperograms at 0.45 V of Pt/C‐cathodic corrosion and commercial Pt/C electrocatalyst in 0.1 M HClO_4_ and 1.0 M CH_3_OH. The current density in the cyclic voltammograms is normalized by the respective mass of Pt loading. Adapted with permission from Ref. [27], Copyright (2018) American Chemical Society.

Besides, cathodic corrosion has been utilized for the fabrication of several metal nanocrystals, including Pt, Pd, Au, Ag, Cu, Rh, Ir, and Ni as well as nanoalloys such as Pt_50_Au_50_, Pd_50_Au_50_, and Ag_*x*_Au_100–*x*_.^[27]^ In this synthesis, poly(vinylpyrrolidone) (PVP) is used as a stabilizer in an electrolyte solution containing non‐reducible cations (Na^+^ and Ca^2+^). Ultrasonication was applied and all bulk wires were subjected to cathodic and anodic treatment at ±10 V using a square waveform at a frequency of 100 Hz. Figure 2a provides a schematic illustration of the nanoparticle fabrication process along with corresponding photographs of the produced nanoparticles. Figures 2b–j display TEM and high‐resolution TEM (HRTEM) images of nanoparticles (Pt, Au, Ag, Pd, Rh, Ir, Cu, and Ni) produced in different electrolytes. The TEM images reveal that these nanoparticles are spherical and uniformly dispersed. Lattice fringes indicating crystallinity are presented in the insets in the upper left corners of the HRTEM images. For instance, the lattice spacing of Ag nanoparticles is assessed to be 2.29±0.05 Å, which closely matches the face‐centered cubic (fcc) Ag lattice spacing of 2.36 Å. Similarly, the determined lattice spacing for Pt (2.245±0.045 Å) aligns with the fcc Pt lattice spacing of 2.25 Å. Moreover, the particle size distributions indicate that Pt, Au−I (produced in Ca(NO_3_)_2_), Au‐II (produced in Na_2_SO_4_), Ag, Pd, Rh, Ir, Cu, and Ni nanoparticles have average diameters of approximately 2.1, 4.4, 9.4, 8.6, 4.5, 6.3, 5.2, 4.7, and 4.2 nm, respectively. Notably, the size of Au−I nanoparticles was approximately half that of Au‐II nanoparticles, revealing that the nature of the cations in the electrolyte influences particle size and morphology. It was believed that the cations impact the metastability of prenucleation clusters formed under negative potentials, thereby affecting the final characteristics of the nanoparticles. The electrocatalytic activity of Pt/C produced through cathodic corrosion (Pt/C−CC) was compared to commercial Pt/C (Pt/C−JM) for methanol electrooxidation. The corresponding values of the electrochemically active surface area (EASA) were estimated as 70.3 and 62.8 m^2^ g^−1^ by integrating the hydrogen desorption charge from −0.2 to 0.1 V *vs*. Ag/AgCl, as shown in Figure 2k. Pt/C−CC exhibits a larger EASA compared to Pt/C−JM, which was attributed to the smaller particle sizes (2.1 nm for Pt/C−CC *vs*. 4.0 nm for Pt/C−JM). Figure 2l demonstrates that the mass activity of Pt/C−CC is nearly 2‐fold higher than that of Pt/C−JM for methanol electrooxidation. Additionally, Pt/C−CC exhibits a higher *j*_f_ / *j*_b_ ratio (where *j*_f_ and *j*_b_ represent the forward and backward peak current densities) compared to Pt/C−JM (Pt/C−CC: 1.5; Pt/C−JM: 0.8), indicating a larger tolerance to CO for Pt/C−CC. This was further confirmed through CO‐stripping experiments (Figure 2m). Both the onset and peak potentials of CO‐stripping for Pt/C−CC (0.44 and 0.56 V *vs*. Ag/AgCl) were more negative than those of Pt/C−JM (0.47 and 0.60 V *vs*. Ag/AgCl), indicating that CO is more easily oxidized at the surface of Pt/C−CC. Furthermore, chronoamperometric curves (Figure 2n) show a slower decay rate for Pt/C−CC, attributed to its higher EASA and greater CO tolerance.

**Figure 3:**
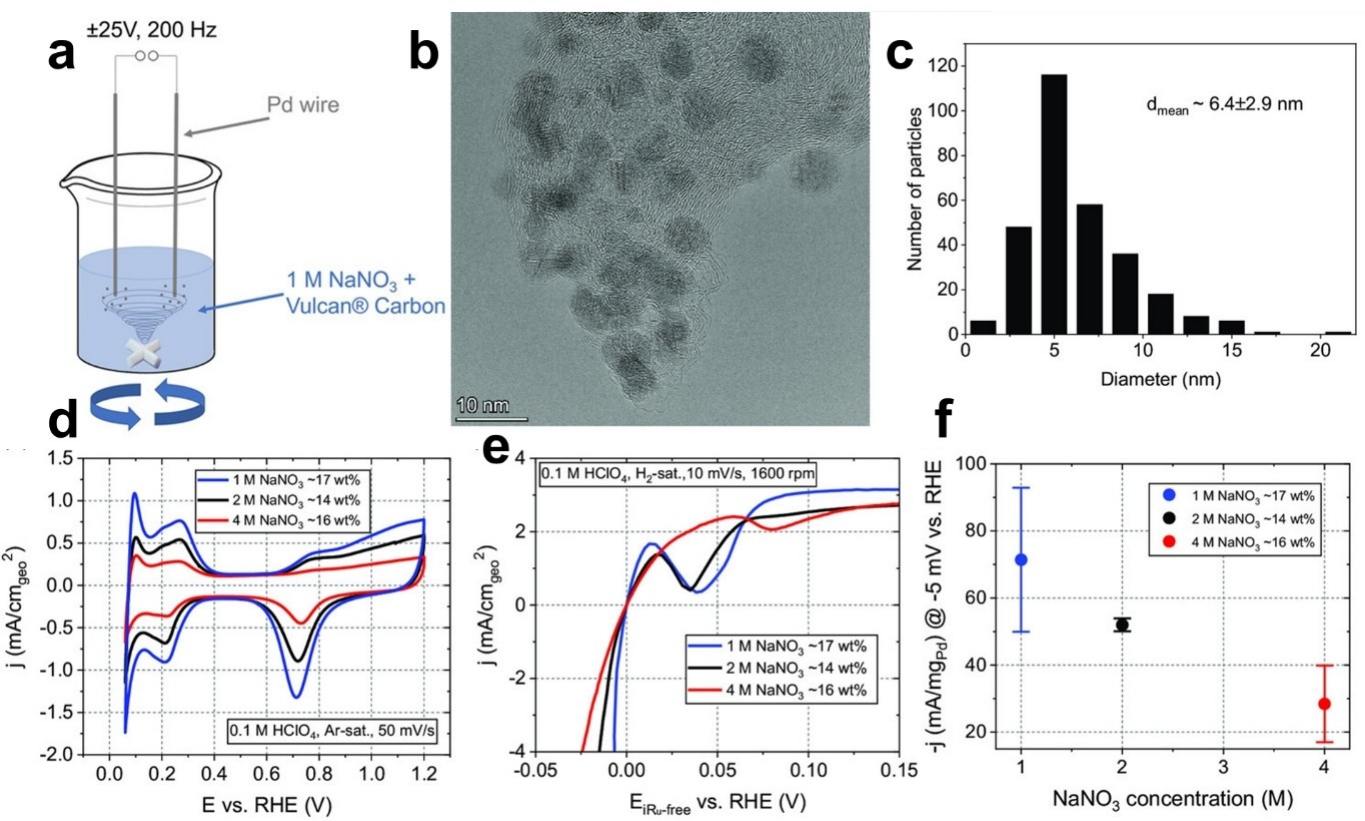
(a) Schematic illustration of the top‐down cathodic corrosion approach. (b) TEM image of the Pd/C catalyst synthesized in 1 M NaNO₃, with (c) the corresponding histogram showing diameter distribution and mean particle size. (d) Cyclic voltammograms of Pd/C catalysts produced in NaNO₃ at varying concentrations (1, 2, and 4 M). Voltammograms were recorded in Ar‐saturated 0.1 M HClO₄ at a scan rate of 50 mV s^−1^ and a rotation speed of 400 rpm. (e) *IR*‐corrected HER polarization curves for Pd/C catalysts, measured in H₂‐saturated 0.1 M HClO₄ at a scan rate of 10 mV s^−1^ and a rotation speed of 1600 rpm. (f) Mass activity comparison at −5 mV vs. RHE as a function of NaNO₃ concentration used in catalyst synthesis. Adapted with permission from Ref. [50], Copyright (2023) Wiley‐VCH GmbH.

A more recent study presents the synthesis of Pd NPs with a diameter below 10 nm using a surfactant‐free cathodic corrosion‐based procedure.^[50]^ The developed method essentially involves a pretreatment of Pd bulk by hydrogen evolution in acidic media (H_2_‐saturated 0.1 M HClO_4_ electrolyte) to form palladium hydride (PdH_*x*_), which causes material embrittlement. Then, the structural properties of Pd wires were even further modified via an annealing and cooling treatment in an Ar atmosphere between 950 and 1050 °C. The annealing step induces the removal of the absorbed hydrogen from the Pd bulk. Moreover, heat treatment with subsequent cooling of Pd formed terrace arrangements and deep grooves or cracks at grain boundaries located on the surface of the Pd wires.[^54^, ^55^] These pre‐treatment steps allowed the corrosion process to evolve more efficiently, producing homogeneously distributed NPs on the carbon support. Three different Pd/C catalysts were produced through cathodic corrosion in 1, 2, and 4 M NaNO_3_ by applying a ± 25 V and 200 Hz sinusoidal potential signal. Figure 3a displays a scheme of the electrochemical setup used to synthesize Pd NPs, which involves multiple parameters that are expected to affect the characteristics of the synthesized Pd NPs. These parameters include the type of wire pretreatment, NaNO_3_ concentration, and the applied corrosion signals, such as frequency and amplitude. Notably, Pd NPs synthesized via a three‐times‐repeated pretreatment step with a ± 25 V, 200 Hz applied sinusoidal voltage signal in 1 M NaNO_3_ electrolyte showed more promising results. The TEM image in Figure 3b demonstrates the homogeneous distribution of the Pd NPs on the Vulcan carbon support, even though certain areas exhibit a slightly higher density of NPs. Figure 3c presents a Pd NP size distribution and a mean average diameter of 6.4 ± 2.9 nm, as measured from TEM and STEM images.

Figure 3d illustrates the cyclic voltammograms of Pd/C catalysts, synthesized using varying concentrations of NaNO_3_ (1, 2, and 4 M) during cathodic corrosion, in Ar‐saturated 0.1 M HClO_4_. Despite minor variations in mass loading (under 3 %), the intensity of hydrogen absorption/adsorption and desorption peaks increases as the NaNO_3_ concentration decreases, paralleling the trend observed in specific surface area (SSA). The SSA demonstrates a near‐linear increase with lower electrolyte concentrations, ranging from a minimum of 27.1 ± 5.0 m^2^ g_Pd_^−1^ for the catalyst synthesized in 4 M NaNO_3_ to 67.3 ± 0.2 m^2^ g_Pd_^−1^ for that produced in 1 M NaNO_3_. This change in SSA is likely to boost the catalyst‘s performance in surface‐sensitive reactions including the hydrogen evolution reaction (HER), as evidenced by the polarization curves in Figure 3e for HER in H_2_‐saturated 0.1 M HClO_4_. These curves display a characteristic peak at slightly positive potentials during the cathodic scan, associated with the formation of PdH_*x*_. Remarkably, the electrochemical assessment of the HER reveals a significant mass activity of 71.4 ± 21.5 mA mg_Pd_^−1^ at −5 mV *vs*. RHE for the Pd/C catalyst synthesized in 1 M NaNO_3_, attributed to the presence of approximately 63 wt.% strained Pd nanoparticles compared to their non‐strained counterparts (Figure 3f).

**Figure 4:**
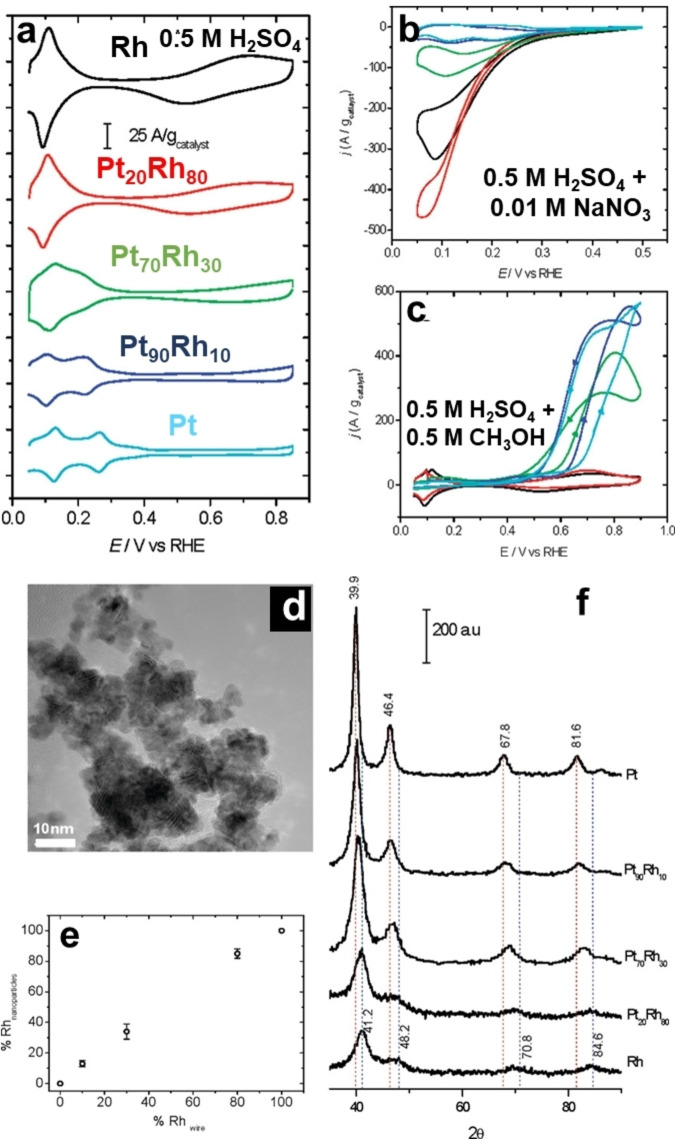
Voltammetric profiles for Pt, Pt_90_Rh_10_, Pt_70_Rh_30_, Pt_20_Rh_80_, and Rh nanoparticles supported on an Au polycrystalline disk in (a) 0.5 M H_2_SO_4_ at 50 mV s^−1^ as well as (b) 0.5 M H_2_SO_4_ + 0.01 M NaNO_3_ solution and (c) 0.5 M H_2_SO_4_ + 0.5 M CH_3_OH solution at 20 mV s^−1^. (d) TEM image of the Pt_90_Rh_10_ nanoparticles. (e) Rh content in the nanoparticles obtained from EDS analysis as a function of the content of Rh on the wire (commercial data). (f) XRD patterns of nanoparticles with various Pt_*x*_Rh. Adapted with permission from Ref. [28], Copyright (2011) American Chemical Society.

Cathodic corrosion was also utilized to fabricate a series of alloy nanoparticles from a bulk alloy electrode.[^26^, ^27^, ^28^, ^56^, ^57^] For instance, Pt−Rh nanoparticles of different compositions were synthesized through cathodic corrosion.^[28]^ Of note, the fabricated alloy nanoparticles retain the same composition and crystal lattice structure of the starting alloy (Figure 4e). Figure 4a illustrates the cyclic voltammetry of the Pt−Rh nanoparticles supported on an Au electrode in H_2_SO_4_ solution. The features observed between 0.05 and 0.4 V *vs*. RHE are connected to the concomitant adsorption/desorption of hydrogen and anions, while those between 0.4 and 0.9 V *vs*. RHE are related to the adsorption of OH species on Rh. By inspecting the peak charges in the hydrogen adsorption region and knowing the mass of the catalyst, the average size of the nanoparticles for pure metals could be estimated. The values obtained through this approximation (Pt ≈9 nm and Rh ≈6 nm) are consistent with those obtained by TEM and XRD (Figures 4d and f). The blank voltammograms exhibit two characteristic features that indicate the presence of bimetallic nanoparticles. As the Rh content is increased, the two hydrogen‐related peaks at lower potentials shift towards more negative potentials, eventually merging into a single peak characteristic of Rh. In the higher potential region, the charge associated with the adsorption/desorption of OH species increases with the concentration of Rh in the sample.

Furthermore, the electrocatalytic activity of the Pt−Rh nanoparticles was assessed for electrocatalytic nitrate reduction and methanol oxidation. Figure 4b shows the voltammograms of nitrate reduction in an acidic solution over PtRh alloy nanocatalysts. Notably, the maximum negative current, which corresponds to nitrate reduction, increases proportionally with the Rh content. Unexpectedly, the Rh_80_Pt_20_ nanoalloy exhibits an even higher current density than pure Rh nanoparticles, which had been known to be the most active electrocatalysts for this reaction. The Rh_80_Pt_20_ sample also exhibits a smaller difference between the reduction currents in the negative and positive scans, suggesting that the reactivity of intermediate species (nitrite, NO) leads to less hysteresis compared to the other samples. Figure 4c shows the voltammetric response of the supported nanoparticles to methanol oxidation. As observed, the Rh and Rh_80_Pt_20_ samples show little to no activity in methanol oxidation due to the strong adsorption of poisoning CO on Rh, which inhibits methanol adsorption, as reported previously. In contrast, electrodes with higher Pt content display significant oxidation currents in the potential range between 0.4 and 0.9 V. The voltammetric profiles of the three high‐Pt‐content electrodes exhibit very similar behavior, with the most significant characteristic being the hysteresis between the positive and negative scans.

## Synthesis of Metal Oxide Nanoparticles

**Figure 5:**
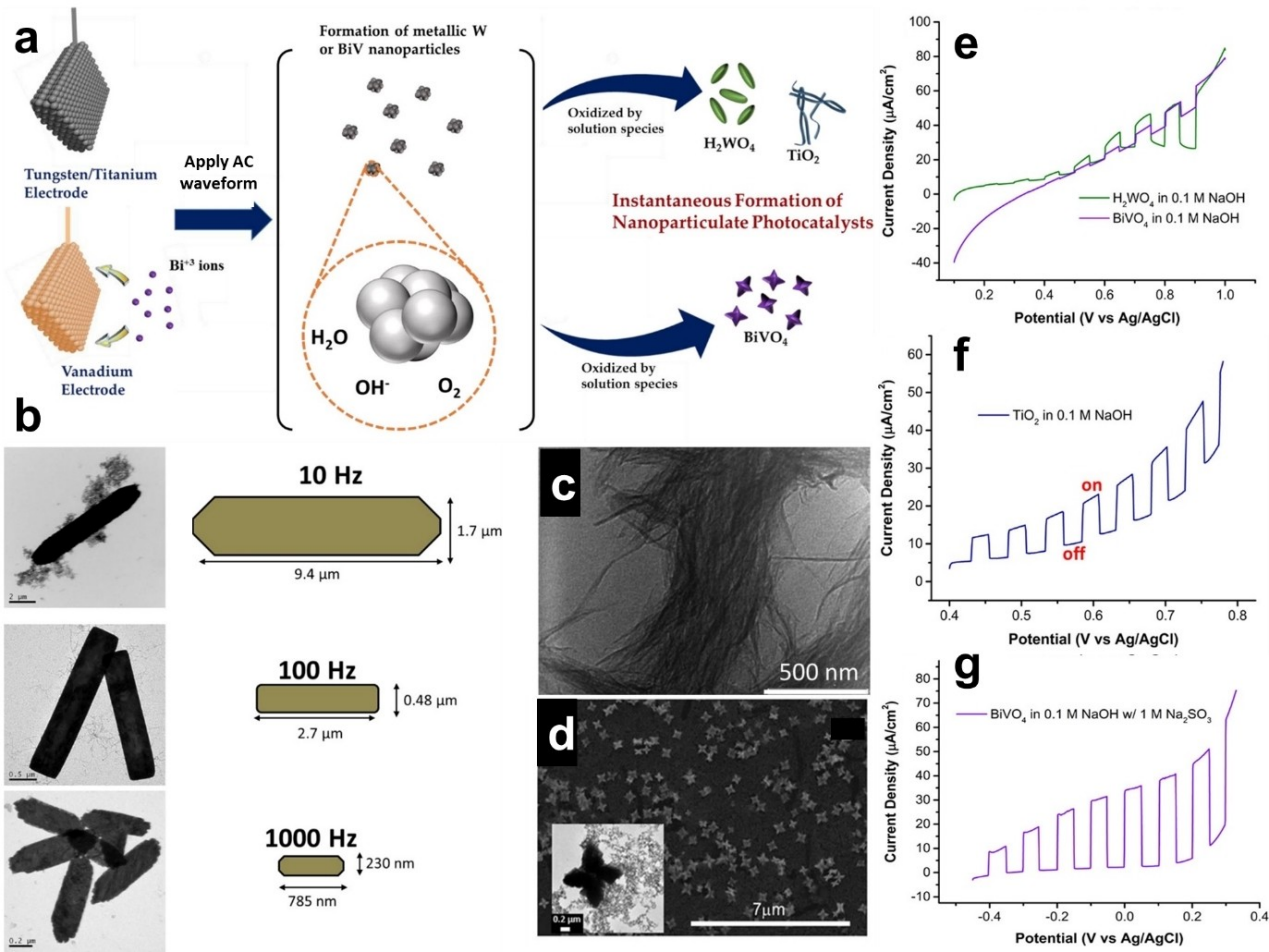
(a) Schematic representation of the cathodic corrosion process used to produce H₂WO₄, TiO₂, and BiVO₄ photocatalysts. (b) TEM images and schematic of H₂WO₄ particles synthesized by cathodic corrosion in a 1 M KHSO₄ solution using a square‐wave voltage between 0 and −10 V at frequencies of 10, 100, and 1000 Hz. (c) TEM image of TiO₂ nanowires fabricated from a Ti wire in 10 M NaOH, using an AC square wave from −10 to 0 V at a frequency of 100 Hz. (d) SEM image of BiVO₄ nanostars, with inset showing an HR‐TEM image. Chopped‐light linear sweep voltammograms for (e) H₂WO₄ and BiVO₄ particles at 100 Hz in 0.1 M NaOH, (f) TiO₂ in 0.1 M NaOH, and (g) the same experiment as (e) repeated for BiVO₄ in 1 M Na₂SO₃. Adapted with permission from Ref. [45], Copyright (2017) American Chemical Society.

Previous studies utilize the cathodic corrosion method to produce suspensions of crystalline core‐shell Sn−SnO_2_ nanoparticles as lithium‐ion battery anode materials and amorphous TiO_2_ nanowires as support for Au nanocatalysts.[^58^, ^59^, ^60^] In another study, the method was extended to produce different oxide nanostructures such as crystalline H₂WO₄ nanorods, TiO₂ nanowires, and crystalline BiVO₄ nanostars (Figures 5a–d).[^45^, ^52^] For the synthesis of H₂WO₄, a tungsten wire was immersed into a 1 M KHSO₄ solution, with a square‐wave voltage applied between 0 and −10 V, and the waveform frequency varied between 10 and 1000 Hz. This study demonstrates that the frequency of the applied square wave significantly influences the size and morphology of the resulting H₂WO₄ structures. Lower frequencies, such as 10 Hz, result in larger, plate‐like structures, while higher frequencies, like 1000 Hz, produce smaller nanorods, as shown in the TEM images (Figure 5b). The authors proposed that the observed variations in H₂WO₄ particle size and shape are driven by the concentration of intermediate species (either anionic or metallic) present in the solution, which are influenced by the frequency and amplitude of the applied waveform. The TiO₂ nanowires were synthesized in a 10 M NaOH solution using an AC voltage cycled between 0 and −10 V at 100 Hz. The produced TiO₂ nanowires have dimensions of approximately 500–800 nm in length and 4–8 nm in diameter, as shown in Figure 5c. For the synthesis of BiVO_4_ nanostars, the vanadium wire was immersed in a CaCl₂ solution, with additional Bi_2_O_3_ to introduce Bi^3+^ ions. A square‐wave voltage oscillating between −8 and +2 V was applied, enabling the formation of complex multimetallic oxide structures (Figure 5d). Among the three oxides, H_2_WO_4_ showed the highest photocatalytic activity for water oxidation (Figure 5e). While still less active than H_2_WO_4_, TiO₂ exhibited noticeable photocurrents (Figure 5f). BiVO_4_ demonstrated limited water oxidation ability (Figure 5e), but showed enhanced activity in the presence of a sacrificial agent, suggesting its utility in applications focusing on the oxidation of other species under visible light as well (see Figure 5g).

**Figure 6:**
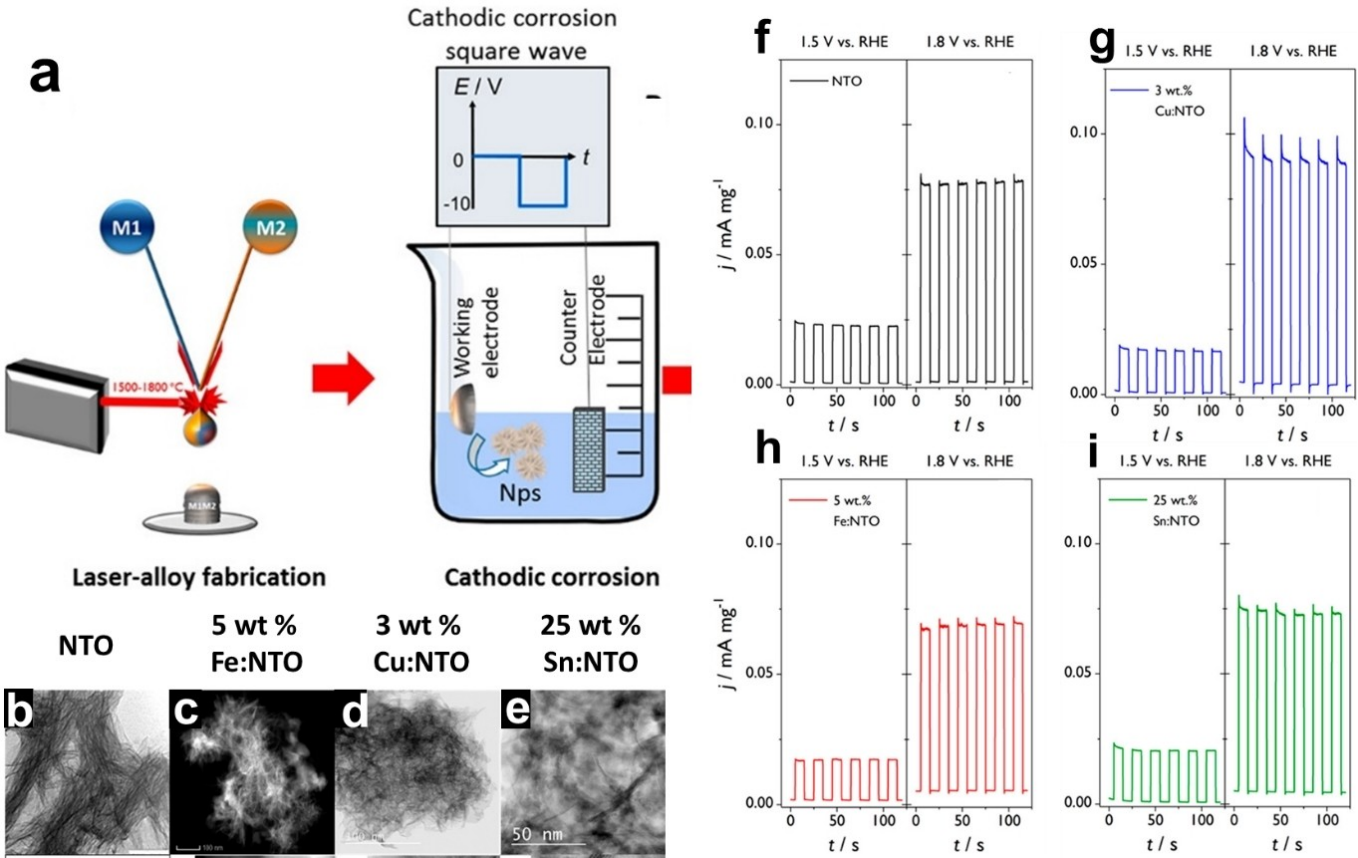
(a) Schematic illustration of alloy and nanoparticle synthesis by combining a laser combinatorial facility with cathodic corrosion. (b) Low‐magnification TEM image of NTO (Niobium Titanate Oxide) nanowires. (c) HAADF‐STEM image of 5 wt.% Fe‐doped NTO, with bright‐field STEM images of (d) 3 wt.% Cu‐doped NTO and (e) 25 wt.% Sn‐doped NTO nanowires. Chopped‐light *I*–*t* curves for titanate nanoparticles synthesized via cathodic corrosion, measured at 1.5 V (*η*≈300 mV) and 1.8 V (*η*≈500 mV) *vs*. RHE in 0.1 M NaOH: (f) NTO, (g) 3 wt.% Cu‐doped NTO, (h) 5 wt.% Fe‐doped NTO, and (i) 25 wt.% Sn‐doped NTO. The potentials correspond to OER overpotentials of approximately 300 and 500 mV, respectively. Adapted with permission from Ref. [61], Copyright (2018) American Chemical Society.

Cathodic corrosion was also employed for the electrochemical fabrication of nanostructured metal‐doped titanates, specifically sodium titanate (Na₂Ti₃O₇, NTO) doped with metals including Fe, Cu, and Sn.^[61]^ This process combined alloy laser fabrication with cathodic corrosion under an alternating current voltage (0 to −10 V) in a 10 M NaOH solution (Figure 6a). The AC voltage facilitated the continuous formation and release of nanoparticles from the electrode surface, producing high‐surface‐area nanostructures with uniformly distributed metal dopants. TEM investigations reveal that these materials form aggregated nanowires, with diameters around 7–12 nm and lengths extending hundreds of nanometers, depending on the dopant type and concentration (Figures 6b–e). The synthesized nanostructures (Na_2_Ti_3_O_7_ (NTO), 25 wt.% Sn:NTO, 5 wt.% Fe:NTO, and 3 wt.% Cu:NTO) were evaluated for their photoelectrochemical (PEC) activity towards the oxygen evolution reaction (OER) under illumination by incident polychromatic white light (UV and visible). The PEC performance varied with the type and concentration of the dopant. For instance, 3 wt.% Cu demonstrates the highest photocurrent for OER, attributed to enhanced visible light absorption and reduced bandgap due to Cu doping (Figures 6f and g). In contrast, Fe and Sn doping do not improve OER performance, with Fe showing increased charge‐carrier recombination and Sn exhibiting surface recombination due to the formation of a passivating SnO₂ shell (Figures 6h and i).

## Synthesis of Single‐Atom Catalysts

In recent years, single‐atom catalysts (SACs), which feature isolated metal atoms anchored by surrounding coordination species of solid supports as active sites, have made remarkable advancements and emerged as highly promising electrocatalysts.^[62]^ Compared to conventional nanoparticle catalysts, the well‐dispersed metal sites in SACs maximize atom utilization, effectively reducing costs while enhancing catalytic performance in both activity and selectivity.^[63]^ The synthesis of SACs follows two primary approaches: the “bottom‐up” and “top‐down” methods.^[64]^ The bottom‐up approach utilizes mononuclear metal complexes as precursors, focusing on achieving atomic dispersion and stabilizing single metal atoms to prevent aggregation. Several strategies are employed, including lowering metal loading to enhance dispersion, constructing coordination sites using nitrogen (N), phosphorus (P), and sulfur (S) atoms to anchor metal species, and utilizing spatial confinement within porous materials such as zeolites, metal‐organic frameworks, and covalent organic frameworks to prevent atom migration and aggregation.[^65^, ^66^, ^67^] Additionally, defect engineering creates vacancies that capture and stabilize single atoms, while controlling thermal motion, such as freezing metal precursors that can further prevent aggregation.^[68]^ On the other hand, the top‐down approach begins with metal nanoparticles or bulk metals, breaking metal‐metal bonds at high temperatures to release single atoms. This process is often facilitated by an oxidizing or ammonia‐rich atmosphere, which generates volatile atomic metal species.[^69^, ^70^] To prevent re‐aggregation, these atomically dispersed metals must be stabilized on supports with abundant coordination sites or defects. Despite their promising properties, SACs face several challenges, including low metal loading in bottom‐up synthesis, which can limit catalytic performance, and the problem of metal atom aggregation, requiring strong interactions between the atoms and supports. Maintaining defect stability is also crucial, as defect reconstruction or elimination can impact catalyst efficiency.[^71^, ^72^] Additionally, precise control over synthesis conditions, such as temperature, solvent type, and pH, is necessary to optimize SAC stability and performance.^[73]^

**Figure 7:**
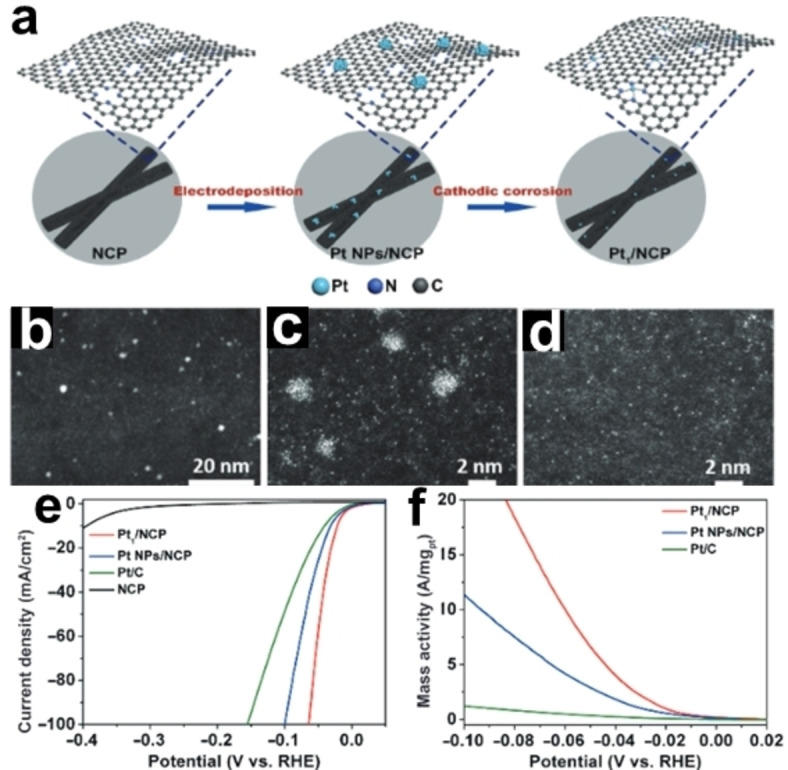
(a) Schematic illustration of the synthesis of Pt_1_/ N‐doped carbon paper (NCP) by cathodic corrosion. HAADF‐STEM images of (b) Pt NPs/NCP, (c) Pt NPs/NCP after cathodic corrosion for 5 min, and (d) Pt_1_/NCP. (e) HER polarization curves, (f) mass activities of Pt_1_/NCP, Pt NPs/NCP, Pt/C, and NCP electrocatalysts. Adapted with permission from Ref. [75], Copyright (2021) SciOpen published by Tsinghua University Press.

Interestingly, cathodic corrosion has been proposed as a facile and powerful approach to synthesize single‐atom catalysts.[^47^, ^74^, ^75^, ^76^] For example, Pt nanoparticles can be transformed through cathodic corrosion into uniformly dispersed Pt single atoms that are immobilized and stabilized by nitrogen coordination sites on N‐doped carbon paper (Pt_1_/NCP), as illustrated in Figure 7a.^[75]^ For synthesizing the precursor of Pt NPs/NCP, electrodeposition was conducted in a solution of 10 mM H_2_PtCl_6_. After this electrodeposition process, the surface of NCP retained the nanoarray structure and the obtained Pt NPs had an inhomogeneous size distribution (Figure 7b). At a high negative voltage of −8 V *vs*. Hg/HgO, Pt NPs were converted into smaller clusters together with single atoms after 5 minutes of cathodic corrosion (Figure 7c). After additional cathodic corrosion for 5 minutes, uniformly dispersed Pt single atoms could be obtained as bright dots in the dark field scanning transmission electron microscopy (HAADF‐STEM) image (Figure 7d). The as‐prepared Pt1/NCP electrodes exhibited a superior electrocatalytic activity towards HER, which is much higher than that of Pt NPs/NCP, Pt/C catalysts, and the NCP substrate, with a low overpotential of 0.022 V at 10 mA cm^−2^ and a low Tafel slope of 28.5 mV dec^−1^ (Figures 7e and f). Long‐term stability under reaction conditions is a crucial factor in evaluating SACs. The stability of Pt₁/NCP was assessed through accelerated durability tests over 5000 cycles and prolonged potential‐constant electrolysis. The polarization curves showed minimal changes after cycling, demonstrating significantly greater stability than Pt/C and Pt NPs/NCP catalysts. SEM and HAADF‐STEM images confirmed that the nanoarray structure of NCP and the isolated Pt atoms remained intact after the tests. Additionally, chronopotentiometric measurements conducted for 100 hours at an overpotential of 0.05 V revealed that Pt₁/NCP retained 97.8 % of its initial current density, whereas Pt NPs/NCP and Pt/C experienced notable degradation (7.87 and 11.7 %, respectively). This exceptional stability is attributed to the robust Pt−N₄ structure that effectively prevents the detachment of active sites. Notably, the proposed cathodic corrosion strategy shows the ability to fabricate other single metal atoms (*e. g*., Pd, Ir, and Cu), which may open a new avenue for metallic SACs preparation.

**Figure 8:**
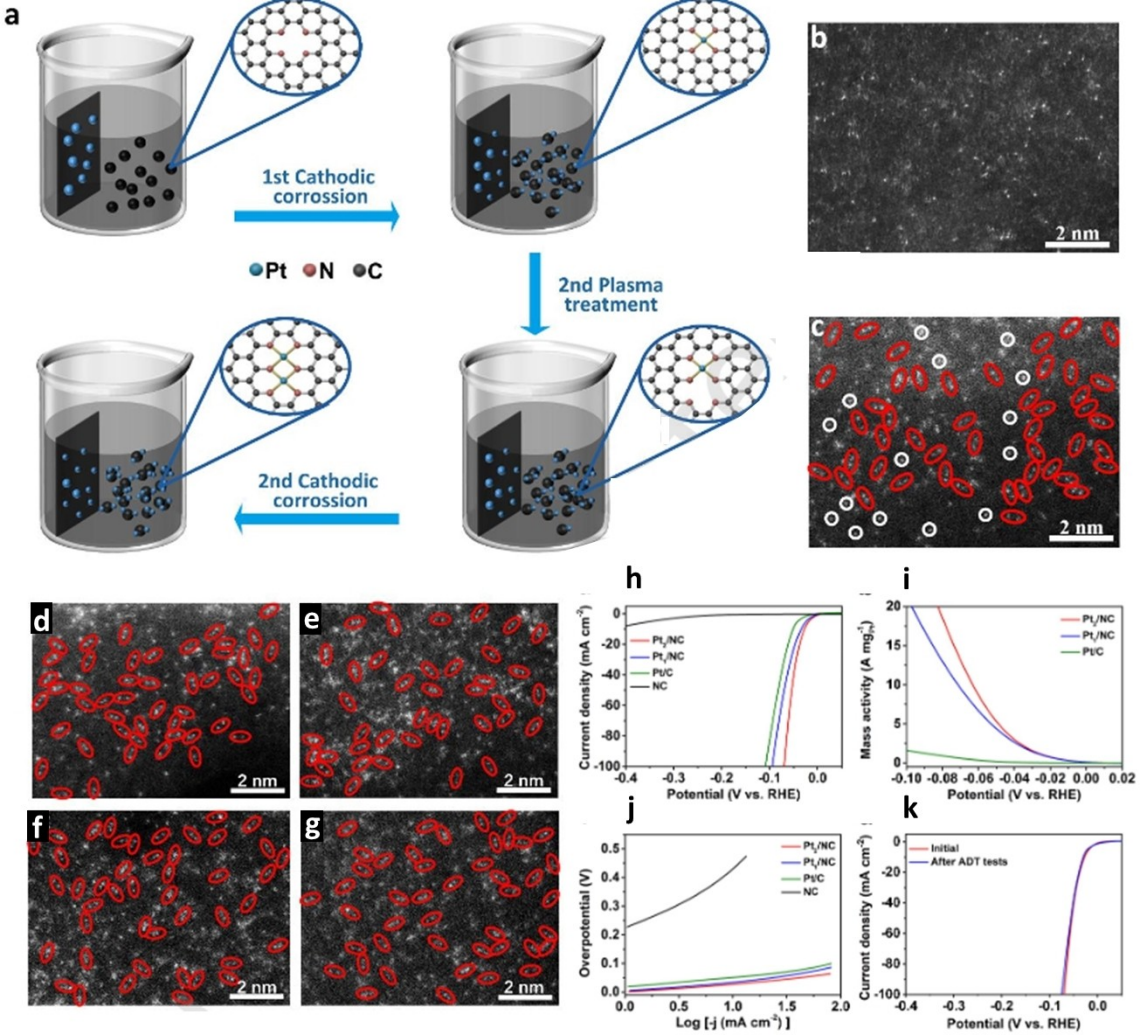
(a) Schematic illustration of the synthesis of Pt_2_/ NC by combining plasma treatment and cathodic corrosion. HAADF‐STEM images of (b) Pt_1_/NC, (c) Pt_2_/NC after one cycle and two cycles of “plasma treatment + cathodic corrosion” (d) Pd_2_/NC, (e) Pt_1_Pd_1_/NC, (f) Pt_2_/NCNT, (g) Pt_2_/NC with large‐scale production. (h) HER polarization curves, (i) mass activities, and (j) Tafel plots of Pt_2_/NC, Pt_1_/NC, Pt/C, and NC electrocatalysts. (k) polarization curves of Pt2/NC before and after accelerated durability tests for 20000 CV cycles. Adapted with permission from Ref. [76], Copyright (2025) SciOpen published by Tsinghua University Press.

More recently, cathodic corrosion has been introduced as a facile method for synthesizing dual‐atom catalysts (DACs) supported on carbon materials.^[76]^ The approach employs a two‐step “plasma treatment + cathodic corrosion” strategy, where metal atoms from nanoparticles (NPs) are etched under a high negative voltage of −5 V *vs*. Hg/HgO in a 5 M KOH electrolyte. During this process, the applied potential breaks down the metallic bonds in the NPs, generating mobile metal anions that diffuse away from the electrode surface. These diffused metal atoms are subsequently captured by nitrogen‐doped carbon substrates, which were pre‐treated with high‐energy nitrogen plasma at 20 kV for 30 minutes to introduce nitrogen‐rich anchoring sites. The first cycle of cathodic corrosion transforms metal NPs into SACs, where the atoms are stabilized by nitrogen coordination. A second round of plasma treatment and cathodic corrosion enables the anchoring of an additional metal atom, forming DACs such as Pt₂/NC (Figure 8a). HAADF‐STEM images confirm the successful formation of Pt₁/NC and Pt₂/NC (Figure 8b and c), verifying the DAC synthesis. The method can also fabricate Pd‐based DACs, such as Pd₂/NC, and heteronuclear DACs like Pt₁Pd₁/NC, by sequentially introducing different metal precursors (Figures 8d and e). The adaptability of this method extends to different carbon substrates such as nitrogen‐doped carbon nanotubes (NCNTs), as demonstrated by the synthesis of Pt₂/NCNT, where Pt dimers are anchored on NCNTs instead of carbon black (Figure 8f). Finally, a scaled‐up synthesis of Pt₂/NC confirms that the method is feasible for large‐scale DAC production, with HAADF‐STEM images showing that Pt₂ dimers remain well‐dispersed even in bulk synthesis (Figure 8g).

The HER polarization curves reveal that Pt₂/NC exhibits the lowest overpotential (0.027 V at 10 mA cm^−2^), outperforming Pt₁/NC, and commercial Pt/C (Figure 8h). Additionally, the mass activity of Pt₂/NC reaches 31.5 A mg^−1^ at 0.1 V, significantly higher than Pt₁/NC (21.4 A mg^−1^) and Pt/C (1.6 A mg^−1^), confirming its superior efficiency (Figure 7i). Tafel slope analysis shows that Pt₂/NC exhibits enhanced HER kinetics (29.9 mV dec^−1^) compared to Pt₁/NC (34.6 mV dec^−1^) and Pt/C (33.3 mV dec^−1^), validating the synergistic interaction of dual‐atom sites in boosting reaction rates (Figure 8j).

Long‐term durability tests confirm the exceptional stability of Pt₂/NC, with negligible performance degradation after 20000 cycles, whereas Pt/C suffers significant loss in activity (Figure 8k). These results highlight Pt₂/NC as a highly efficient and durable HER catalyst, demonstrating the potential of DACs for practical hydrogen production applications.

## Conclusions and Outlooks

Cathodic corrosion represents an adaptable and efficient approach to fabricating a wide array of nanoparticles and single‐atom catalysts, offering noteworthy merits over conventional methods in terms of speed, simplicity, and scalability besides the cleanness of the produced nanoparticles. By empowering precise control over particle size, morphology, and composition, this method can be utilized for tailoring catalyst characteristics, and thus performance to specific applications in energy conversion and storage, and beyond. The future of cathodic corrosion‐based nanoparticle synthesis is proposed to focus on expanding the range of materials beyond metals and metal oxides to include carbides, nitrides, and phosphides, which have potential applications in catalysis and energy storage. To achieve this, more in‐depth mechanistic studies are essential, as they can provide enhanced control over the synthesis process. Efforts should be directed towards scaling up cathodic corrosion methods for industrial applications, ensuring reproducibility and efficiency in large‐scale nanoparticle production. In summary, tailoring the electronic and surface properties of nanoparticles through controlled corrosion parameters could enable the rational design of highly efficient electrocatalysts, photocatalysts, and sensors.

## Conflict of Interests

The authors declare no conflict of interest.

4

## Biographical Information

*Mohamed M. Elnagar obtained his Ph.D. (with distinction) from the Institute of Electrochemistry, Ulm University, Germany, and is currently a postdoctoral fellow at the same institute. His research spans electrocatalysis, electroanalytical chemistry, photocatalysis, and plasma catalysis. With numerous high‐impact publications, he has made significant contributions to fundamental electrochemical processes, including cathodic corrosion and metal electrode stability. His work integrates experimental and analytical techniques to advance energy conversion and sustainable catalytic technologies*.

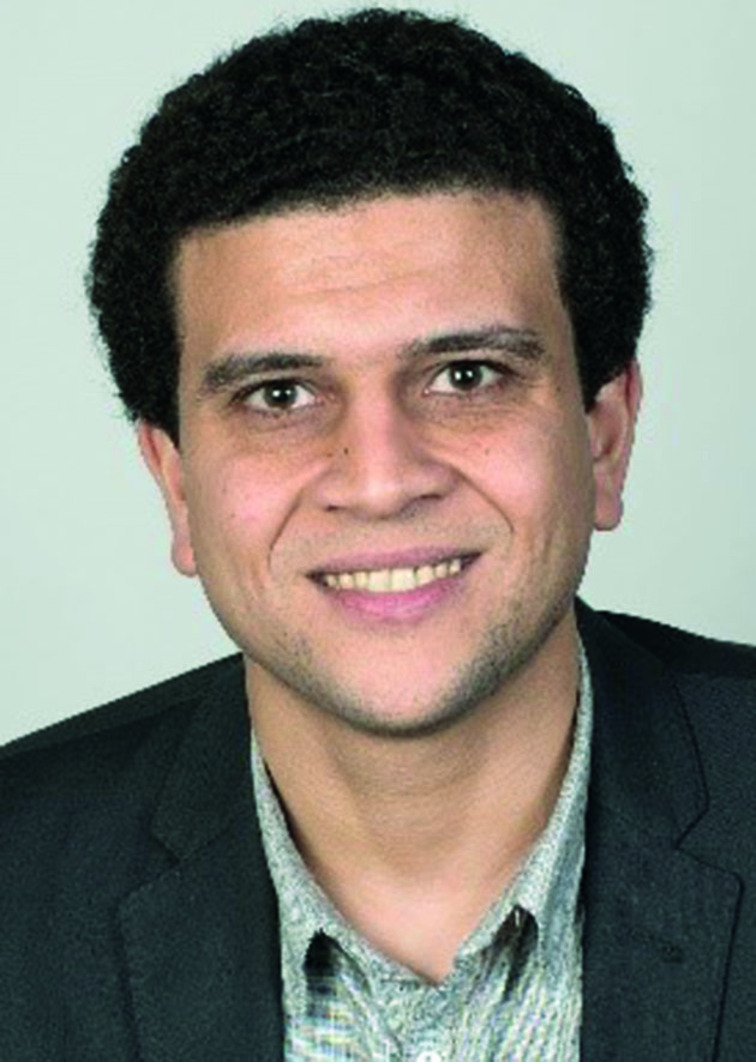

## Biographical Information

*Ludwig Kibler is a Senior Research Scientist at the Institute of Electrochemistry at Ulm University, Germany. He received his Diploma and PhD degrees under the supervision of Prof. Dieter M. Kolb. He is active as an academic in the physical chemistry laboratories as well as in electrochemistry and physical chemistry courses. His main research interests in fundamental research are single crystal electrochemistry, metal deposition, electrocatalysis, and tailoring of electrode materials and nanostructures. Current investigations involve the kinetics of surface reconstruction phenomena, surface oxidation and surface restructuring employing electrocatalytic probe reactions*.

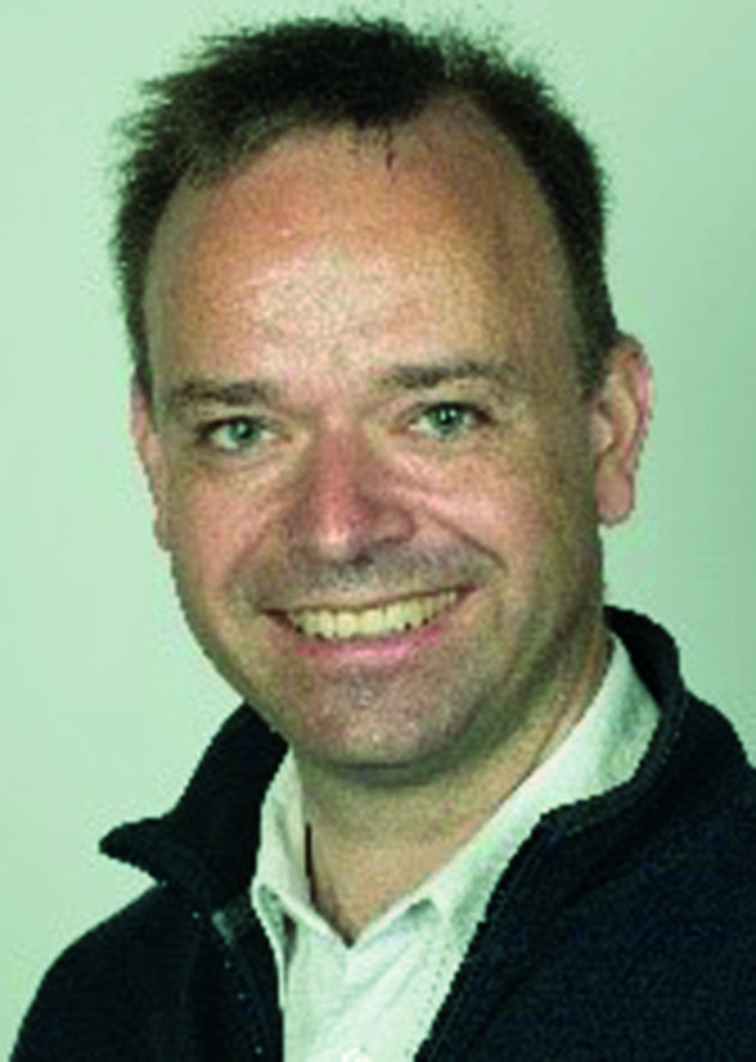

## Biographical Information

*Timo Jacob studied physics at the University of Kassel, receiving his PhD in Feb. 2002. After a two‐year postdoctoral stay at Caltech, in 2004 he joined the theory department of the Fritz‐Haber‐Institute in Berlin, and finished his habilitation in theoretical physics at the Free University Berlin in Nov. 2008. In 2007 he became leader of an independent Emmy‐Noether research group at Ulm University and received an ERC‐Starting Grant in 2010. Since 2011 he is director of the Institute of Electrochemistry at Ulm University, where his research covers the fundamentals of electrochemical interfaces and processes both experimentally and theoretically*.

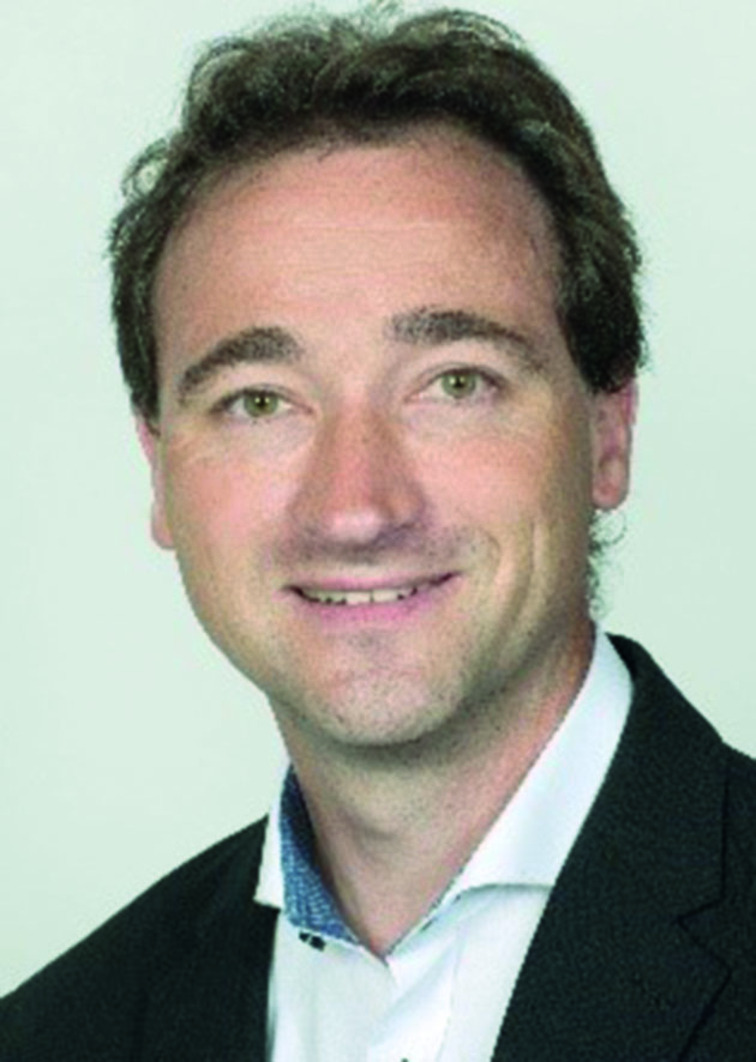

## Data Availability

The data that support the findings of this study are available from the corresponding author upon reasonable request.
